# Self-perceived loneliness on cognitive functioning and on self-perceived cognitive abilities in aging

**DOI:** 10.3389/fmed.2026.1725659

**Published:** 2026-03-05

**Authors:** Sonia Montemurro, Radu Valentin Camenita, Giulia Sebastianutto, Massimo Nucci, Sara Mondini

**Affiliations:** 1 Department of Philosophy, Sociology, Education and Applied Psychology (FISPPA), University of Padua, Padua, Italy; 2 Department of General Psychology, University of Padua, Padua, Italy; 3 Human Inspired Technology - Research Centre HIT, University of Padua, Padua, Italy; 4 Servizi Clinici Universitari Psicologici (SCUP) - Centro di Ateneo, Padua, Italy; 5 Department of Social and Developmental Psychology (DPSS), University of Padua, Padua, Italy; 6 IRCSS San Camillo Hospital, Venice, Italy

**Keywords:** aging, cognitive performance, loneliness, mental health, self-perception

## Abstract

**Introduction:**

Loneliness in older adults is recognized as a psychosocial factor influencing cognitive performance. This study examines the impact of self-perceived loneliness both on actual cognitive performance and on individuals’ self- perceived cognitive abilities.

**Methods:**

A sample of 510 healthy older adults (354 females) aged between 64 and 103 years (mean = 78.78±8.72) was studied. Participants’ self-perceived loneliness was assessed on UCLA Loneliness Scale-3 (Hughes et al., 2004), their cognitive performance with the standardized cognitive screening GEMS (Global Examination of Mental State) and their self-perceived cognitive efficiency with SMAC (Sclerosi Multipla Autovalutazione Cognitiva [Multiple Sclerosis cognitive self-evaluation]).

**Results:**

Results showed that the higher the perceived loneliness, the worse the cognitive performance (B = -1.18; *p* < 0.001) and the lower the self-perceived cognitive efficiency (B = 2.48; *p* < 0.001). However, loneliness is associated with a tendency to underestimate one’s own cognitive abilities also when performance is average (*F* = 9.75, *p* < 0.001).

**Discussion:**

Loneliness in older adults should be regarded as highly impactful not only on cognitive performance but also on perception of one’s own abilities. Indeed, even when cognitive efficiency is well preserved, loneliness leads to underestimation of personal cognitive resources.

## 1 Introduction

Italy, by 2051, will have 280 elderly for every 100 young people, an increase in age-related chronic diseases, including neurocognitive disorders (1). Therefore, research has been focusing on modifiable risk factors that can slow cognitive decline.

The Lancet Commission on dementia (2) outlined 14 potentially modifiable risk factors (i.e., low education, hearing loss, high LDL cholesterol, depression, traumatic brain Injury, physical inactivity, diabetes, smoking, hypertension, obesity, excessive alcohol, social isolation, air pollution and visual loss). Overall, they have been estimated to account for 45% of dementia cases globally, suggesting that many diagnoses may be preventable.

The focus of the present study is to examine perceived loneliness within the broader phenomenon of social isolation, considering loneliness as a construct potentially related to social isolation among older adults. While social isolation refers to physical separation from others (3), loneliness pertains to the subjective perception of being disconnected from others (4). Specifically, loneliness is a distressing condition characterized by a perceived lack of closeness, either quantitative and/or qualitative (5). Older adults are particularly vulnerable to loneliness, a condition that is influenced not only by health issues such as chronic illnesses, but also by social and environmental circumstances such as living alone and loss of friends or partners. Loneliness in later life is associated with a range of negative health outcomes, including impaired immune function, increased risk of mental health disorders, and cardiovascular disease. These findings highlight loneliness as a significant biopsychosocial risk factor for poor cognitive functioning (6).

In a systematic review article, Cardona and Andrés (7) showed that perceived loneliness, measured on the UCLA Loneliness Scale-3, is significantly associated with degree of cognitive decline in older adults. Du et al. (8) in a cohort of 4,772 older persons aged over 50, showed that higher levels of loneliness (measured on the UCLA Loneliness Scale-3) were associated with lower scores in global cognitive functioning. The authors also highlighted the mediating role of leisure activities in this relationship (e.g., including reading, writing, attending senior citizen university, playing chess, poker, or mahjong, and doing crafts), suggesting their ability to reduce the negative effect of loneliness. Another study showed the relationship between loneliness (measured on the UCLA Loneliness Scale-3) and physical frailty (measured with Fried phenotype of physical frailty) in older adults, confirming in a sample of 2,817 people aged ≥60 that people who experienced high levels of loneliness were at increased risk of becoming physically frail (9).

A narrative review of Guarnera et al. (10) summarized the current understanding of how loneliness and social isolation may influence cognitive aging and its potential links with dementia. The authors confirmed a significant association between loneliness, social isolation, and reduced cognitive function in older adults with the contribution of underlying neural and physiological mechanisms (e.g., alterations in cortisol secretion and changes in brain structure like gray and white matter, hippocampal volume).

Loneliness is recognized to affect cognition, but little is known on its effect on self-perception of cognition. Only in very few studies the relationship between self-perceived loneliness and self-perceived cognitive efficiency has been explored. Pecchinenda et al. (11) for example showed that individuals with higher levels of perceived loneliness showed subjective higher working memory decline. However, to date, it is still not clear to what extent the degree of loneliness perceived by older adults may contribute to self-perception of being more or less cognitively efficient while performing a formal assessment.

Several studies have addressed the topic of self-perception of general decline in aging. For example, Hu and Li (12) examined the relationship between self-perception of aging, by addressing memory and the mediating role of social connections. They reported that loneliness, rather than social disconnectedness, was associated with the perception of aging. In another study, “subjective aging” was demonstrated to play a significant role in explaining and predicting cognitive aging and decline in older adults [see Fernández-Ballbé et al. (13) for a systematic review].

A relationship between perceived loneliness, self-perceived cognitive efficiency, and global cognitive performance in tests in older adults can therefore be hypothesized. Our aim is to investigate the relationship between self-perceived loneliness, cognitive functioning and self-perceived cognitive efficiency in a population of older adults.

## 2 Methods

The study was conducted in accordance with the Declaration of Helsinki and it was approved by the Ethical Committee of the School of Psychology of the University of Padua (Code: 446-b, approval date: 04/03/2024).

### 2.1 Participants

A sample of 510 healthy participants (354 females, 156 males), aged between 64 and 103 years (M = 78.78; SD = 8.72), with between 1 and 27 years of education (M = 10.91; SD = 4.92) was recruited across different regions of Italy. The perceived Socio-Economic Status of the participants (on a scale between 1 and 6) averaged 3.55 (SD = 0.97, range = 1–6). Participants were well distributed across socio-demographic variables and also across different occupations (see Supplementary Table S1).

The inclusion criteria were: age 64 or older and being either a native Italian speaker or having lived in Italy for at least the previous 20 years. Participants with psychiatric disorders or neurological diagnoses (such as stroke or traumatic brain injury) were excluded from our study.

Participants were recruited from five-day centers for the elderly and four homes for the elderly. Data were collected by examiners who had received standardized training in several sessions conducted by the same instructor. The training included reviewing the study materials and hands-on practical exercises. Each examiner was also given a standardized protocol to follow throughout the study.

### 2.2 Materials

The following four instruments were administered to measure different dimensions: (a) the UCLA Loneliness Scale-3 (three-item version) (14, 15) for self-perceived loneliness; (b) the self-perception of cognitive functioning questionnaire (SMAC, Sclerosi Multipla Autovalutazione Cognitiva, [Multiple Sclerosis cognitive self-evaluation]; (16)) for self-perception of cognitive functioning, and (c) the Global Examination of Mental State (GEMS) (17) to verify objective cognitive performance. The short version of the Cognitive Reserve Index questionnaire (sCRI-q) (18) was also administered to evaluate the influence of cognitive reserve, in particular the richness of our participants’ intellectual life and in general their life-style.

- The UCLA Loneliness Scale-3 (14, 15) is a three-item questionnaire that measures three dimensions of loneliness: relational connectedness, social connectedness and self-perceived isolation. The final score ranges from 3 to 12, with 12 indicating a very high perception of loneliness.
- The Self-Perception of Cognitive Functioning Questionnaire (Sclerosi Multipla Autovalutazione Cognitiva, SMAC) (16) aims at exploring participants’ self-perception of cognition. The 25-item questionnaire investigates how often the participant feels they encounter cognitive difficulties in everyday living. The final score ranges from 0 to 100, with 100 indicating low self-perceived cognitive efficiency. For statistical analyses, the raw score of the SMAC was reverse-coded so that SMAC and GEMS scores had the same direction toward efficiency/deficiency (e.g., higher scores indicated better cognitive functioning and greater perceived cognitive efficiency).
- The Global Examination of Mental State (GEMS) (17) is a brief paper-and-pencil cognitive screening test measuring global cognitive functioning. The administration takes around 10 min to complete, and the psychometric properties of the test allow avoidance of the ceiling effect. The final score ranges from 0 to 100, with 100 indicating high global cognitive functioning.
- The short Cognitive Reserve Index questionnaire (sCRI-q) (18) is a semi-structured interview which collects and quantifies the main cognitively stimulating activities a person has carried out during their adult life. The questionnaire provides a final composite score obtained from the investigation of three main domains: education, working activity and leisure time activities. The estimated Cognitive Reserve Index then classifies the person from a low to a high level of cognitive reserve.

### 2.3 Procedure

All participants were informed of the general aim of the study and signed an informed consent before beginning the experimental procedure. Participants were individually administered the described instruments in a fixed order to control for potential order effects. The entire procedure lasted approximately 45 min, with breaks provided upon the participant’s request.

### 2.4 Statistical analyses

Statistical analyses were performed using R software (version 4.4.2; 26). First, both descriptive statistics and multivariate analyses were performed to examine the role of perceived loneliness and self-perception of cognitive efficiency in determining cognitive performance. Subsequently, a standardized discrepancy score was computed to capture individual tendencies to overestimate or underestimate their cognitive abilities. Specifically, SMAC and GEMS scores were standardized, and SMAC scores were reverse-coded by multiplying the corresponding z-scores by −1. This transformation ensured that, for both measures, higher scores reflected greater perceived cognitive efficiency and performance, respectively. The discrepancy was calculated as follows:

$$\text{Standardized discrepancy}=({\text{zSMAC}}^{*}-1)-\text{zGEMS}$$

Positive values indicate a tendency to overestimate one’s cognitive abilities, whereas negative values reflect underestimation. To examine associations between discrepancy scores and relevant predictors, correlation analyses and a one-way ANOVA were carried out.

## 3 Results

The UCLA Loneliness Scale-3 scale reported a mean score of 6.13 (SD = 2.39), while the SMAC questionnaire showed a mean score of 25.48 (SD = 13.72). The distribution of GEMS scores showed a negative asymmetry, reflecting a prevalence of high scores among participants. The mean score of GEMS was 73.94 (SD = 16.37) with scores ranging from 17.5 to 100 (see Table 1).

**Table 1:** Descriptive statistics of observed scores on the UCLA Loneliness Scale-3 (14, 15).

Descriptives	Min	Max	Mean	SD
UCLA-3	3	12	6.13	2.39
SMAC	1	75	25.48	13.72
GEMS	17.5	100	73.94	16.37

SMAC, Self-Perception of Cognitive Functioning Questionnaire [Sclerosi Multipla Autovalutazione Cognitiva (16)]; GEMS, Global Examination of Mental State (17).

### 3.1 Loneliness and cognitive efficiency

The correlation analysis between perceived loneliness and cognitive efficiency measured with GEMS showed a weak and negative correlation (*r* = −0.16). We tested three linear models with cognitive efficiency as the dependent variable, subsequently incorporating Age and UCLA Loneliness Scale-3 in a combined model. Based on Akaike Information Criterion (AIC) comparisons, the combined model including Age and UCLA scores demonstrated the best fit, accounting for 21% of variance in cognitive efficiency (R^2^ = 0.21; see Table 2). This result showed that the more frequently older adults reported feeling lonely, the worse they performed in the GEMS cognitive screening.

**Table 2:** Linear regression model including global examination of mental state (GEMS) (17) as the dependent variable and Age and UCLA Loneliness Scale-3 (14, 15) as covariates.

GEMS				
Model R^2^ = 0.21	Estimate	Std error	*t* value	*p-*values
Intercept	145.19	6.14	23.65	< 0.001
Age	−0.81	0.074	−10.98	< 0.001
UCLA-3	−1.18	0.27	−4.38	< 0.001

### 3.2 Loneliness and self-perceived cognitive efficiency

The correlation analysis showed a moderate positive correlation (*r* = 0.43) between perceived loneliness and self-perceived cognitive efficiency measured with SMAC. We then tested three linear models with self-perceived cognitive efficiency as the dependent variable, incorporating Age and UCLA Loneliness Scale-3 into a combined model. Based on Akaike Information Criterion (AIC) comparisons, the model including UCLA Loneliness Scale-3 scores showed the best fit, accounting for 19% of variance in self-perceived cognitive efficiency (R^2^ = 0.19; see Table 3). This result showed that the more frequently older adults reported feeling lonely, the less they reported feeling globally cognitively efficient in their daily lives.

**Table 3:** Linear regression model including self-perception of cognitive functioning questionnaire [Sclerosi Multipla Autovalutazione Cognitiva, SMAC; (16)] as the dependent variable and UCLA Loneliness Scale-3 (14, 15) as the covariate.

SMAC				
Model R^2^ = 0.19	Estimate	Std error	*t* value	*p-*values
Intercept	10.27	1.51	6.80	< 0.001
UCLA-3	2.48	0.23	10.80	< 0.001

### 3.3 Loneliness and standardized discrepancy

The discrepancy score reflects the standardized difference between self-perceived cognitive efficiency (SMAC) and objective cognitive performance (GEMS). For example, a score of −0.5 indicated a tendency to underestimate one’s cognitive abilities. Whereas, a score of +0.5 indicated a tendency to overestimate one’s cognitive abilities.

**Figure 1:**
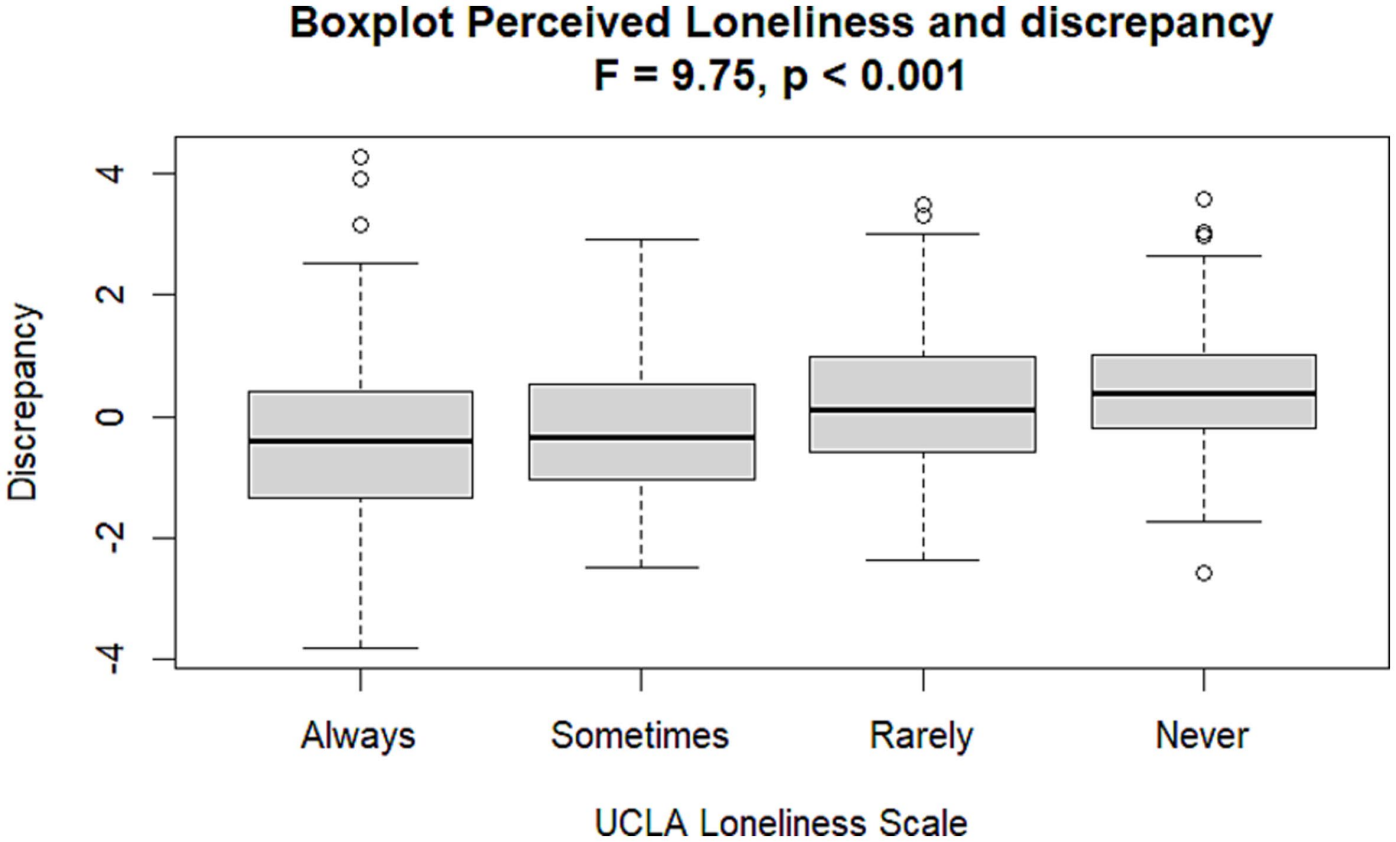
Distribution of the discrepancy index (Discrepancy Cog). The values below zero indicate underestimation of one’s cognitive efficiency; 0 indicates correspondence between cognitive efficiency on GEMS and self-perception of cognitive efficiency on SMAC; values above 0 indicate overestimation of one’s own cognitive abilities.

Results show that the standardized discrepancy had a median score of −0.11 and a low positive asymmetry, reflecting a prevalence of individuals underestimating their cognitive abilities (See Figure 1).

**Figure 2:**
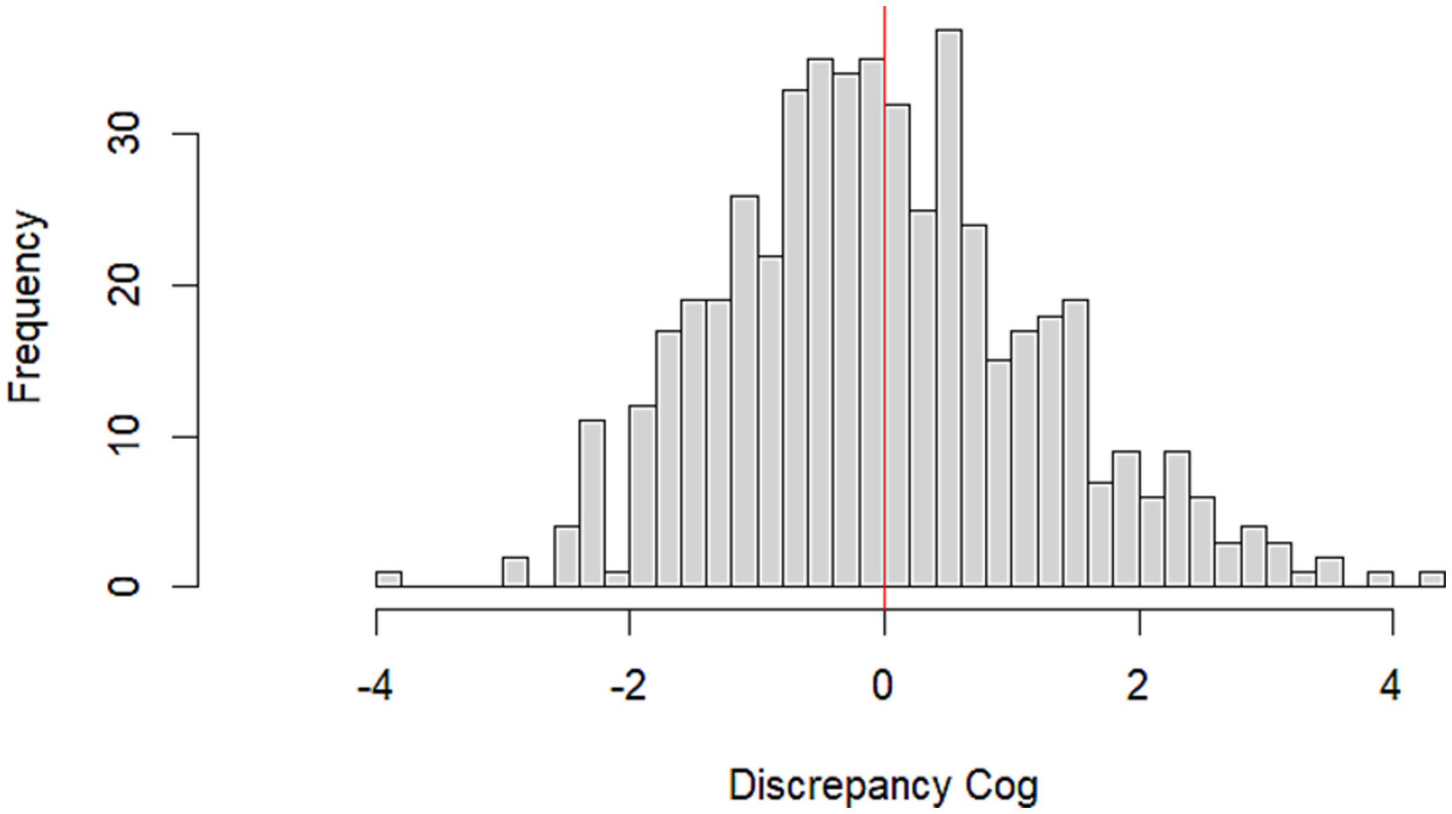
Boxplot illustrating the distribution of discrepancy scores across four levels of perceived loneliness. Loneliness was categorized in quartiles of the UCLA Loneliness Scale-3: Always lonely, sometimes lonely, rarely lonely, and never lonely. Analyses revealed a significant difference in discrepancy scores across groups, with higher loneliness levels associated with greater underestimation of cognitive efficiency.

A negative correlation was observed between discrepancy scores and perceived loneliness (*r* = −0.22). To further investigate the role of loneliness in shaping the discrepancy between cognitive efficiency and its self-perception, participants were categorized into four groups based on the quartiles of their loneliness scores: Always Lonely (8–12), Sometimes Lonely (6–8), Rarely Lonely (4–6), Never Lonely (3–4). A one-way ANOVA revealed a significant difference in discrepancy across the groups (*F* = 9.75, *p* < 0.001; See Figure 2). This result showed that the more frequently older adults reported feelings of loneliness, the greater the discrepancy between their objective performance on GEMS and their self-perception of cognitive efficiency on SMAC. Specifically, those reporting higher levels of loneliness were more likely to underestimate their cognitive efficiency.

## 4 Discussion

This research aimed at investigating how self-perceived loneliness relates to cognitive functioning and perceived cognitive efficiency among older adults.

We examined the role of perceived loneliness in modulating the extent to which older adults are able to maintain an accurate self-perception of their own cognitive abilities. Understanding this relationship is important as discrepancies between cognitive performance and self-perception of cognition may have significant implications for mood and quality of life.

The issue of loneliness in aging populations is particularly relevant, especially within the Western cultural context where we collected our data. In this world older adults and their possible experiences of loneliness are often regarded as outdated, sometimes socially reinforcing “ageism” phenomena [stereotypes, prejudice and discrimination toward others or oneself based on age - definition of the World Health Organization; see also (19)], thus perpetuating social exclusion rather than fostering intergenerational interaction. By contrast, in collectivistic cultures older individuals are more often considered as custodians of knowledge and cultural heritage.

Loneliness only partially overlaps social isolation, yet they are separate concepts. While loneliness refers to self-perception of being disconnected from others and may arise when an individual’s social needs are not adequately met (4), social isolation can be related to a more objective physical separation from others, typically assessed by size of social network and frequency of social interactions (3).

In a recent review, Puyané et al. (20) have shown a significant prevalence of loneliness and social isolation among community-dwelling European older adults, with risk factors including for example health deterioration or loss of social networks. These authors highlighted that addressing the loneliness challenge of aging populations requires an integrative approach able to embrace individual, relational and contextual factors. Loneliness is often conceptualized as a negative subjective experience arising from the absence of meaningful relationships. Although sometimes it is semantically overlapped with social isolation, the two concepts are quite different, especially because social isolation represents an objective condition that could sometimes be a deliberate choice.

With this research we have confirmed that loneliness affects cognitive functioning and self-perception of cognitive efficiency, and this aligns with previous research (21, 22). Such a result indicates that self-perceived loneliness may play a role in cognitive decline, and longitudinal studies are needed to further confirm that the rate of perceived loneliness is itself a factor related to the risk of dementia.

Few studies have directly examined the modulatory role of perceived loneliness in older adults’ ability to self-reflect on their own cognitive efficiency. In this study we also found that the loneliest older adults are, the higher is their tendency to underestimate their cognitive efficiency. These results align to some extent with existing literature by Cacioppo and Hawkley (23) and Holwerda et al. (24), who advanced the hypothesis that loneliness may involve prolonged activation of the hypothalamic–pituitary–adrenal (HPA) axis, resulting in high cortisol levels that link stress to cognitive deterioration.

Taking together our findings and results reported in the literature, the perceived loneliness could be considered as a potential predictor of mental health outcomes. This idea is also supported by past findings by Holahan and Holahan (25) who showed that self-efficacy is not only positively associated with social support over a one-year period, but it also significantly relates to the risk of depression, acting as a direct protective effect against depression.

One possible limitation of this study is the gender disproportion in our sample, which had a very high number of women. This disparity reflects the difficulty in recruiting males willing to participate in the experimental protocol. In fact, among the people we contacted, women appeared to be more interested and motivated to participate in a research study on aging. Future studies should strive to obtain a more balanced gender distribution to allow for a more precise examination of potential gender-related differences.

Another limitation relates to the way the sample was recruited. Our participants mostly belonged to day centers and four homes for the elderly, where individuals typically enjoy strong social networks, actively engage in community activities, and interact frequently with peers.

Future research should consider recruiting participants from healthcare facilities, hospitals, and private clinics to ensure a more heterogeneous representation of psychosocial profiles.

Further limitations related to the instruments used should be acknowledged.

We are aware that GEMS is a cognitive screening tool rather than a comprehensive neuropsychological assessment; however, its use was considered appropriate because it has recently undergone validation and standardization and shows strong psychometric properties. We decided to use it because the sample consisted of healthy individuals and the study did not aim at exploring specific cognitive domains in depth. Although the 3-item version of the UCLA Loneliness Scale was chosen to minimize respondent fatigue in an elderly sample, it provides less detailed information than the original longer version and might therefore reduce sensitivity. Finally, SMAC is limited by the lack of available normative data and the absence of published evidence from large population samples, which restrict its generalizability and broader applicability.

By addressing these limitations in future research, a deeper understanding of the interactions among relevant factors could be achieved, thus providing a more accurate representation of older adults and fostering the development of targeted tools and interventions for those most in need.

## 5 Conclusion

This study provides important insights into the impact of loneliness on older adults, not only on their ability to perform cognitive tasks, but also on perception of their own efficiency and their capacity to make objective evaluations of their cognitive functioning. Our findings indicate that older adults who experience greater loneliness are more prone to cognitive decline, perceive themselves as less effective, and tend to underestimate their cognitive abilities. In other words, even when cognitively competent, lonelier older adults may experience mood disturbances that affect their perceived efficiency, with clear implications for their quality of life, particularly regarding mood and overall mental health. These results highlight the importance of promoting greater social integration for older adults, recognizing them as valuable resources rather than burdens.

## Data Availability

The raw data supporting the conclusions of this article will be made available by the authors, without undue reservation.
