# Supplementary material for: Self-perceived loneliness on cognitive functioning and on self-perceived cognitive abilities in aging
Source: Front Med (Lausanne). 2026 Mar 5;13:1725659. doi: 10.3389/fmed.2026.1725659 (PMC13000764; doi:10.3389/fmed.2026.1725659)
Supplement: Supplementary file 1 [file Data_Sheet_1.pdf]

SUPPLEMENTARY MATERIAL

Self-perceived Loneliness on Cognitive functioning and on Self-perceived Cognitive abilities in Aging

Montemurro S[1]., Camenita R.V.[1], Sebastianutto G[1]., Nucci M.[2], Mondini S[1,3,4,5]

[1]Department of Philosophy, Sociology, Education and Applied Psychology (FISPPA), University of Padua, Italy

[2]Department of General Psychology, University of Padua, Italy

[3]Human Inspired Technology - Research Centre HIT, University of Padua, Italy

[4]Servizi Clinici Universitari Psicologici (SCUP) - Centro di Ateneo;

[5]Department of Social and Developmental Psychology (DPSS), University of Padua, Italy

[4]IRCSS San Camillo Hospital, Venice, Italy

Key-words: Loneliness, Aging, Cognitive performance, Self-perception

Table S1. Distribution of occupational positions reported by the participants. Job titles are presented in Italian using the masculine generic form, regardless of the participant's gender.

	Working Activity (English)	Working Activity (Italian)	Frequency
1	Accountant	Ragioniere/Commercialista	5
2	Agent	Rappresentante	4
3	Agricultural Worker	Bracciante agricolo	12
4	Architect	Architetto	1
5	Armed forces (police inspector, warrant officer, military personnel, police officer, air-force officer, army officer, municipal police officer)	Forze dell'ordine (Ispettore polizia, maresciallo, militare, poliziotto, sottufficiale aeronautica, ufficiale esercito, vigile urbano)	12
6	Art Restorer	Restauratore di dipinti	1
7	Assistant Cook	Addetto mensa	2
8	Attendant	Usciere	1
9	Attorney (tax office)	Procuratore (ufficio imposte)	1
10	Babysitter	Babysitter	1
11	Baker	Panettiere	2
12	Bartender	Barista	2
13	Beautycian	Estetista	1
14	Biologist	Biologo	1
15	Blacksmith	Fabbro	1
16	Care assistant	OSS	3
17	Cashier	Cassiere	1
18	CEO	Amministratore delegato	4
19	Civil Servant	Funzionario	4
20	Cleaners and helpers	Addetto alle pulizie	6
21	Cloakroom attendant	Guardarobiere	2
22	Cobbler	Calzolaio	3
23	Construction Worker	Muratore	2
24	Consultant	Consulente	2
25	Cooks	Cuoco	4
26	Corporate Liquidator	Liquidatore aziendale	1
27	Dentist	Odontoiatra	1
28	Dietitian	Dietista	1
29	Drivers (bus, truck)	Autista	3
30	Editor	Editore	1
31	Educator	Educatore	3
32	Electrician	Elettricista	1
33	Engineer	Ingegnere	4
34	Entrepreneur (agriculture, IT, construction, pastry)	Imprenditore (agricolo, ditta informatica, edile, pasticcere)	11
35	Executive Manager	Direttore aziendale	5
36	Fisiotherapist	Fisioterapista	3
37	Foreign correspondent	Corrispondente estero	1

38	Forestry workers	Tagliatore legna	1
39	Freelancer	Libero professionista	2
40	Front desk clerk	Sportellista	1
41	Hairdresser	Parrucchiere	5
42	Handicraft worker	Artigiano	5
43	Healthcare Assistant	Assistente sanitario	2
44	Homemaker	Casalinga	19
45	Housekeeper	Governante	1
46	Ice Cream Maker	Gelataio	2
47	Interpreter	Interprete	1
48	Ironer	Stiratrice	1
49	IT Specialist	Informatico	4
50	Labor Union Activist	Sindacalista	2
51	Laundry worker	Lavandaia	1
52	Lawyer	Avvocato	2
53	Librarian	Bibliotecario	2
54	Lithographer	Litografo	2
55	Manager	Responsabile (amministrativo, aziendale)	18
56	Manufacturing Labourer (food industry worker, over operator, steelworker, textile worker)	Operaio (alimentare, addetto forni, siderurgia, tessile)	41
57	Mechanic	Meccanico	2
58	Mechanical Designer	Progettista meccanico	1
59	Medical doctor	Medico	5
60	Midwife	Ostetrica	1
61	Miner	Minatore	1
62	NA	NA	3
63	Never Worked	Non ha lavorato	11
64	Nurse	Infermiere	12
65	Office Clerk (Municipal Clerk, Human Resources Clerk, Postal Clerk, Administrative Clerk, Accounting Clerk)	Impiegato (comunale, risorse umane, poste, amministrativo, contabile)	75
66	Painter	Pittore	1
67	Pharmacist	Farmacista	2
68	Photographer	Fotografo	3
69	Plumber	Idraulico	1
70	Postal Worker	Portalettere	1
71	Priest	Parroco	1
72	Printing trades worker	Tipografo	2
73	Psychologists	Psicologo	1
74	Publisher	Presidente casa editrice	1
75	Real estate agents	Agente immobiliare	2
76	Researcher	Ricercatore	1
77	Sales Assistant	Commesso	12
78	Sales representatives	Agente commerciale	3
79	School Janitor	Collaboratore scolastico	5

80	School Principal	Dirigente scolastico	2
81	Seamstress	Magliaia	3
82	Shopkeeper/Merchant	Commerciante	12
83	Social Worker	Assistente sociale	4
84	Specialised Manufacturing Labourer	Operaio specializzato	6
85	Speech Therapist	Logopedista	2
86	Tailor	Sarto	18
87	Teacher (all levels)	Insegnante (scuola materna, primaria, secondaria)	71
88	Technical Expert	Perito	1
89	Technician (agricultural, appliance, IT, health and safety)	Tecnico (agricolo, elettrodomestici, informatico, prevenzione)	6
90	Train Driver	Macchinista treni	1
91	Train Manager	Capostazione ferroviario	2
92	Turist assistant	Assistente turistico	1
93	Typist	Dattilografo	1
94	University Professor	Professore universitario	1
95	Upholsterer	Tappezziere	1
96	Waitress	Cameriere	2
97	Warehouse Worker	Magazziniere	2
98	Writer	Scrittore	2
